# Study on Transient Contact-Impact Characteristics and Driving Capability of Piezoelectric Stack Actuator

**DOI:** 10.3390/s20010233

**Published:** 2019-12-31

**Authors:** Yanze Li, Yunian Shen, Qiaoping Xing

**Affiliations:** Department of Mechanics and Engineering Science, School of Science, Nanjing University of Science and Technology, Nanjing 210094, China; liyanze@njust.edu.cn (Y.L.); xingqp@njust.edu.cn (Q.X.)

**Keywords:** piezoelectric stack actuator, impact, contact force, transient responses, coefficient of restitution

## Abstract

The transient contact-impact mechanism and driving capability of the piezoelectric stack actuator is analyzed using both experimental and theoretical methods. An experimental setup and its corresponding measurement approaches for the transient responses are designed. The launch range of the object resulting from the first contact-impact is measured through laser doppler vibrometer and the motion process is captured by high-speed camera. Experimental results illustrate that the launch range increases firstly and decreases subsequently as the frequency of the sine driving voltage increases. Meanwhile, considering the local viscoelastic contact deformation, a theoretical methodology including the mechanics model for the driving process is proposed. Based on the Lagrange equations of second kind, the governing equation of the driving system is derived. Transient responses are calculated using the fourth-order Runge–Kutta integration method. Contact forces and Poisson’s coefficient of restitution are calculated by the proposed theoretical method. The results of launch range show that the theoretical solutions have a good agreement with the experimental data. The peak value of contact force increases firstly and decreases subsequently with the increase of voltage frequency. In addition, the coefficient of restitutions is roughly 0.9 when *f* is greater than 3.5 kHz.

## 1. Introduction

Piezoelectric actuators have been widely used in the fields of precision positioners with high-strain materials, multilayer device designs, and mass fabrication processes for portable electronic devices, ultrasonic motors for micro-robotics, and smart structures [1,2,3,4,5]. In addition to using the inverse piezoelectric effect of intelligent material, many relevant applications are also designed based on the structural contact-impact characteristics such as piezoelectric stack actuator (PSA, made in Tokyo, Japan) during the droplet formation of a liquid micro-dispenser [6], new piezoelectric driving quadruped micro-robot [7], novel impact rotary motor [8], and vibration excitation system equipped with PSA [9]. The contact-impact is a transient dynamics problem with unilateral constrain. It will cause contact deformation at the local contact zone, high amplitude contact force, and high-frequency vibration of the rest of body. In addition, the contact force usually cannot be measured directly. Hence, there is no strongly effective tool to analyze the driving capability of PSA.

Several contact-impact experiments of PSA have been performed. Liu et al. [10] adopted the experimental method to analyze the precision position of a block. The results show that a high-precision and large-stroke positioning can be obtained by a table consisting of the piezoelectric element with a spring and a hammer. Pozzi and King [11] designed an experimental setup of PSA to drive an object. However, the existed experimental work still lacks a systematica analysis on the impact responses. In addition, some scholars focused on theoretically predicting the contact-impact responses of PSA. A series of dynamic models and methods for the impact actuator were also developed. Carrera et al. [12,13] used the finite element method to analyze the electro-mechanical properties of multilayered piezoelectric structures based on the classical equivalent singlelayer (ESL) model and advanced layer-wise (LW) model. The solutions of ESL, LW, and mixed ESL/LW models are compared. Adriaens et al. [14] presented an electromechanical piezo model where a first-order differential equation was adopted to describe the hysteresis effect, and a partial differential equation was derived to describe the mechanical behavior. Jia et al. [15] discussed using uniform strain model and Bernoulli–Euler model to analyze PSA. Liu et al. [16] computed the dynamic responses of a soft-mounted lead zirconate titanate (PZT) actuator using a distributed parameter model. The dynamic equation of the system was derived by the finite element method. Huang and Tan et al. [17] considered the effect of hysteresis and the nonlinear dynamics of a piezoelectric stack was analyzed. Shen and Yin [18] proposed a dynamic substructure model to discuss a similar actuator. More accurate dynamic responses were obtained in their work. However, these models are inefficient because they have a large number of computational degrees of freedom.

In this paper, the transient contact-impact mechanism and driving capability of the PSA is analyzed using both experimental and theoretical methods. The launch range of the object (*L*_r_) is measured in the experiment. In addition, the theoretical solution of the contact force and the coefficient of restitution (COR) are obtained through contact-impact model. In Section 2, the experiment on the object driven by the contact-impact of PSA is performed. The variation of the driving capability is discussed as driving voltages change. The transient responses of the object are monitored directly via a laser doppler vibrometer (LDV, made in Karlsruhe, Germany). In Section 3, a theoretical methodology with a contact-impact dynamic model for analyzing driving capability of PSA is formulated. The viscoelastic contact law is accounted for treating contact constraint. The governing equations are derived based on the Lagrange equations of second kind. The fourth-order Runge–Kutta integration method is utilized to solve the equations. Finally, the contact force and the coefficient of restitution are calculated with the theoretical method proposed in Section 3.

## 2. Contact-Impact and Driving Experiment

The PSA studied in this paper consists of multi-layered ceramics, which are made of polycrystalline piezoelectric materials. The piezoelectric effect results from the linear electromechanical interaction between the mechanical and electrical states in crystalline materials with no inversion symmetry. The piezoelectric effect is a reversible process: materials exhibiting the piezoelectric effect (i.e., the internal generation of electrical charge resulting from an applied mechanical force) also exhibit an inverse piezoelectric effect (i.e., the internal generation of a mechanical strain resulting from an applied electrical field). The inverse piezoelectric effect refers to when an electric field is applied in the polarization direction of piezoelectric elements and these piezoelectric elements produce mechanical deformation or mechanical pressure in a certain direction. When the applied electric field is removed, this deformation or pressure disappears as well.

Based on the inverse piezoelectric effect, a piezoelectric ceramics stack will produce a periodic deformation continuously if a corresponding current is applied on it. During such a process, any object which contacts with one end of the PSA can be bounced away. This contact-impact characteristic is utilized to drive a steel object.

### 2.1. Experiment Setup

**Figure 1:**
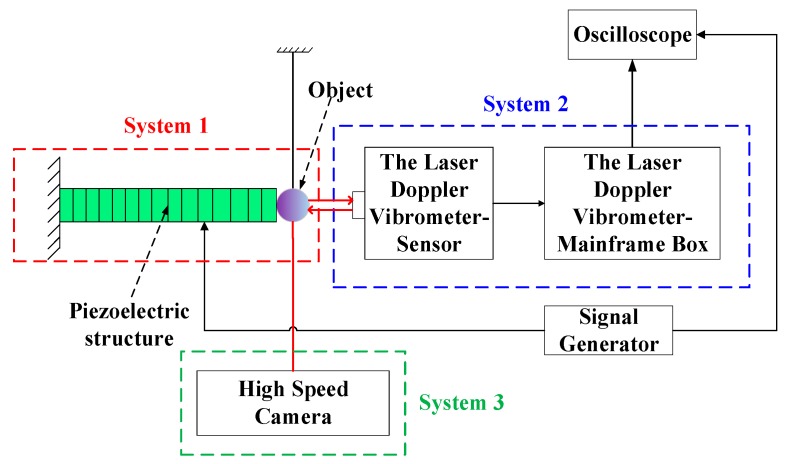
Experimental setup for the contact-impact driving process.

Figure 1 shows the schematic diagram of the experimental system. An experimental table to achieve the contact-impact phenomenon of PSA is designed and established. Additionally, the corresponding measurement systems are designed for the contact-impact test of PSA in order to measure *L*_r_.

The whole experimental setup is divided into three systems as illustrated in Figure 1. System 1 is the driving mechanism of an object (i.e., steel ball) impacted by PSA. System 2 is the LDV to detect the displacement of the object. System 3 is the high-speed camera (made in Wuhan, China) which is used to catch the transient photos of the motion process. Some system parameters of experimental setup are listed in Table 1.

PSA, the object, the experimental table, and the driving voltage generator are included in the system 1. PSA, 5 × 5 × 20 mm in size, has a limitation of 150 V voltage, and wires are welded on both sides of it. During the experiment, one end of the PSA is consolidated with a fixed wall using cyanoacrylate glue whose structural formula is CH2 = C(CN)-COO-C2H5 (Deli company, Ningbo, China, model number 7174). The other end of the PSA is free. The driving voltage is applied on the PSA. The object is vertically suspended beside the free end of the PSA, contacting with the center of the PSA’s cross section exactly. According to the principle of the inverse piezoelectric effect, the deformation of the PSA under the driving voltage can drive the object away.

**Figure 2:**
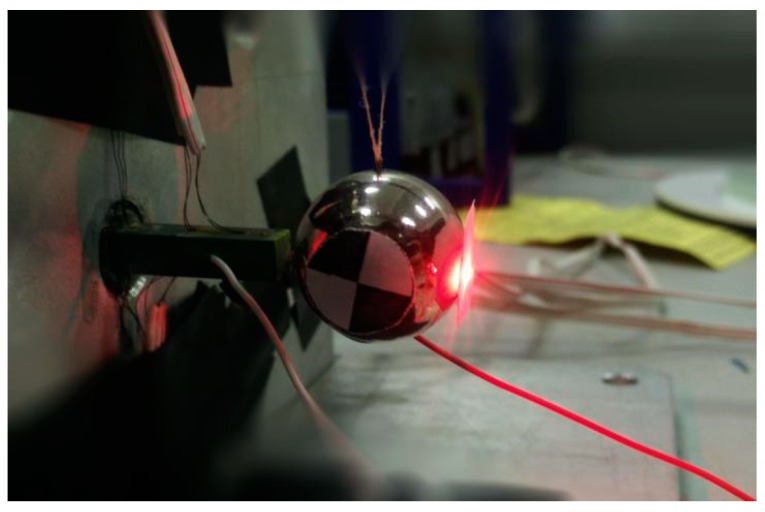
The steel object driven by a piezoelectric stack actuator (PSA).

Meanwhile, the displacement of the object over time is measured by the LDV. Figure 2 is a photo of its transient recorded process. The sensitive paper used by the LDV and the speckle paper used by the high-speed camera are stuck on the object. The red light on the right is from the LDV during the experiment.

**Figure 3:**
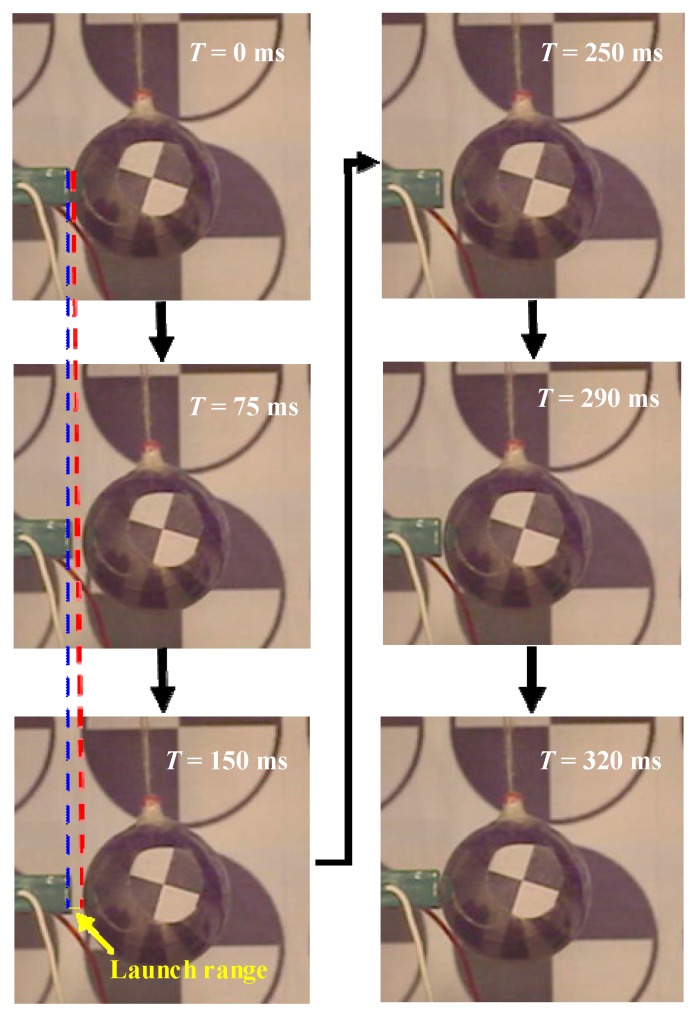
Transient pictures taken by high-speed camera.

Figure 3 shows one driving progress of the object taken by high-speed camera. To demonstrate the bouncing phenomenon clearly in the image, the *L*_r_ we chose in Figure 3 was much larger than that which resulted from the first contact driving. With the high-speed camera taking 2000 pictures per second and 640 × 480 pixels for each picture, the driving process is recorded. The position of the object can be illustrated by the pixels’ position of its center pixel. In Figure 3, the distance between two pixels is 1.52 mm. The displacement of the object $X_{2}$ can be calculated by the number of the pixels that the center of the speckle paper crosses.

### 2.2. Analysis of Experimental Results

During the experiment, it can be found that *L*_r_ strongly depends on the load conditions. Hence, the relationship between *L*_r_ and the amplitude *V*_a_ of the driving voltage is studied. Moreover, a comprehensive analysis of the influence of the other factors on *L*_r_ is done, which includes the frequency *f* of the driving voltage or the wave form of the driving voltage. Table 2 is the experimental scheme for these analyses. To analyze this problem systematically, six groups of experiments (i.e., hundred times of experiment) are performed, and the corresponding dynamic responses of the object during the driving experiment are recorded. The specific experiment data are illustrated in Table A1, Table A2, Table A3 and Table A4 in Appendix A.

**Figure 4:**
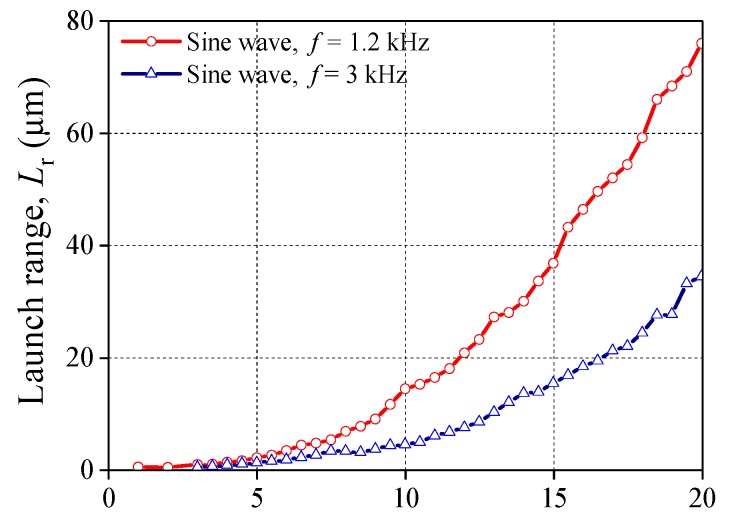
Relationship between *V*_a_ and *L*_r_ (groups 3 and 4).

The relationship between *L*_r_ and *V*_a_ is shown in Figure 4. The driving voltage is chosen as a sine wave with *f* being 1.2 kHz and 3 kHz, respectively. From Figure 4, it can be found that under the action of the driving voltage, *L*_r_ increases monotonously as *V*_a_ increases from 1 V to 20 V once *f* and the wave form of the driving voltage are certain. *L*_r_ under the voltage of 1.2 kHz (see red line) are generally greater than that of 3 kHz (see blue line).

**Figure 5:**
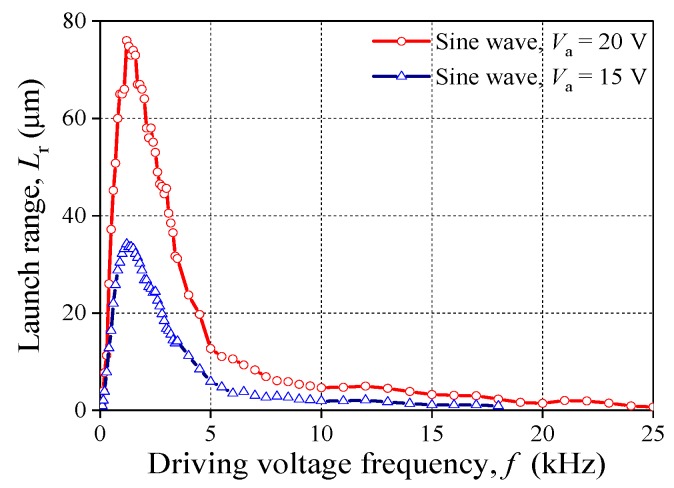
Relationship between *f* and *L*_r_ (groups 1 and 2).

In order to detect the influence of *f* on *L*_r_, the driving voltage as sine wave and its amplitude are maintained. As shown in Figure 5, *L*_r_ increases as *f* increases before 1.2 kHz. On the contrary, *L*_r_ decreases with the increasing of *f* when *f* > 1.2 kHz; but the decrease rate is less than the increase rate. Eventually, *L*_r_ drops down and approaches 0 μm when *f* > 2.0 kHz. In addition, the peak value of the curve is 76 μm and 34.2 μm for the case of 20 V and 15 V, respectively.

**Figure 6:**
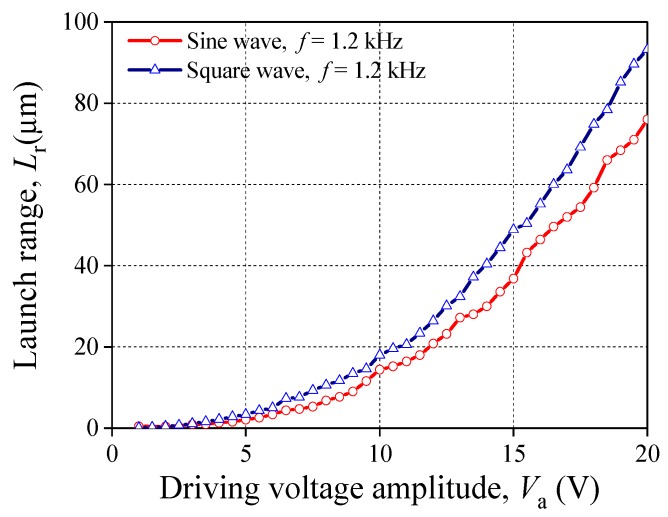
Relationship between *V*_a_ and *L*_r_ (groups 3 and 5).

**Figure 7:**
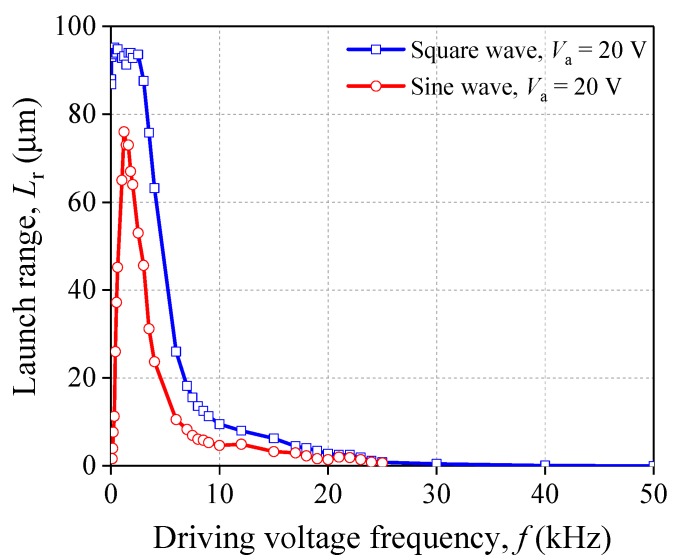
Relationship between *f* and *L*_r_ (groups 1 and 6).

Furtherly, the waveform of the driving voltage also has a key role which effects the change rule of *L*_r_. Setting the driving voltage into a square wave, we collected the data of *L*_r_ under different *V*_a_ or *f*. Then they are compared with that under the sine wave. The experimental results are plotted in Figure 6 and Figure 7. In Figure 6, when the PSA is acted by the square wave and *f* is fixed, the tendency of the relationship between *L*_r_ and *V*_a_ is consistent with that under the sine wave. However, the driving ability of PSA applied by the square wave is stronger than that under sine wave. Even though the two kinds of wave forms have the same voltage amplitude, the relationships between *f* and *L*_r_ are obviously different (see Figure 7). For the square wave, *L*_r_ maintains as constant, about 94 μm when *f* < 3 kHz. Then, *L*_r_ declines when *f* > 3 kHz. Eventually, *L*_r_ drops and approaches 0 μm.

## 3. Theoretical Methodology and Contact-Impact Model

**Figure 8:**
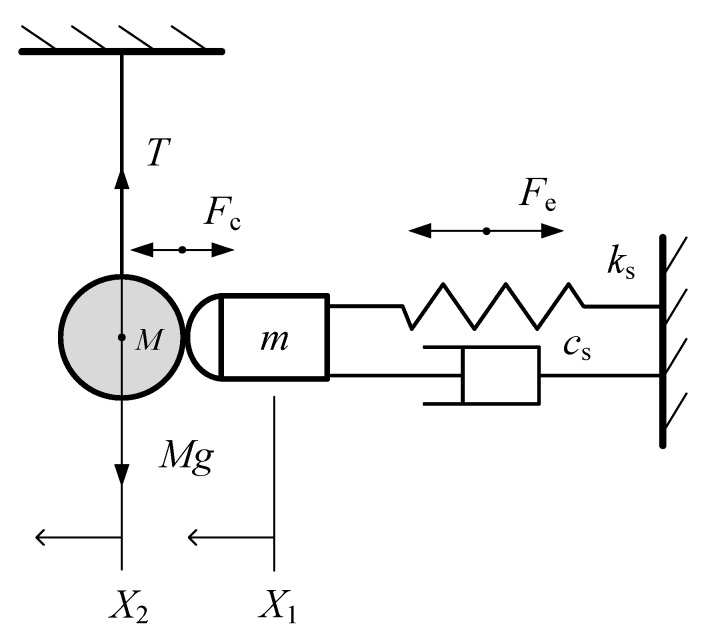
Dynamic model of the driving system.

Due to the limitation of the experimental methods, some key parameters which are paramount at the time of the actuator design, such as contact force or coefficient of restitution, cannot be measured directly. Hence, in order to reveal these impact characteristics during the contact-impact process, a theoretical method including a dynamic model (see Figure 8) is proposed. Considering the addition–deletion of the contact constraints, the contact-impact event studied in Section 2 should be assumed to consist of three phases:

Pre-impact: PSA and the object are at rest. They are contacting with each other, but there is no contact force because contact penetration between them does not exist during this phase.

Contact-impact: Acted by the driving voltage, the transient deformation of PSA occurs. PSA is in contact with the object. A contact constraint is added.

Post-impact: PSA vibrates only under the driving voltage. There are no constraints on it. The object body is bounced away and performs a single pendulum motion.

### 3.1. Modeling of PSA

Figure 8 is the dynamic model of the driving system in which the PSA is simplified as a mass-spring-damping system. Generally, to consider the effect of the hysteresis, the method similar to that of [17] can be used. A state variable (i.e., system nonlinear disturbance [17]) can be introduced into the piezoelectric force *F*_e_. Based on the Lagrange equations of second kind, the governing equation of the mass-spring-damping system without contact constraint is (i.e., during the pre-impact) derived as follows:

(1) $$m{\ddot{X}}_{1}=-c_{s}{\dot{X}}_{1}-k_{s}X_{1}+\left(F_{e}-k_{s}F_{0}\right)$$

where m is the mass of PSA. $X_{1}$ is the displacement of the mass block.$X_{1}$ also represents the axial extension or compression deformation of PSA. $k_{s}$ and $c_{s}$ are the equivalent stiffness coefficient and structural damping coefficient, respectively. $F_{e}$ is the piezoelectric force which depends on the voltage. $F_{0}$ is the state variable (i.e., system nonlinear disturbance [17]) brought by the effect of hysteresis. Ha et al. [19] also proved that hysteresis behavior can be described as energy dissipation, and it will decrease the piezoelectric displacement. In our paper, to make Equation (1) become simpler, the effect of hysteresis is assumed to be neglected. Furtherly, the governing equation can be expressed as:

(2) $$m{\ddot{X}}_{1}=-c_{s}{\dot{X}}_{1}-k_{s}X_{1}+F_{e}$$

To eliminate the error brought by this assumption, a larger damping is used to dissipate the energy which should be dissipated by the effect of hysteresis indeed. Like this, the acceptable displacements of the PSA also can be predicted by the simper Equation (2).

**Figure 9:**
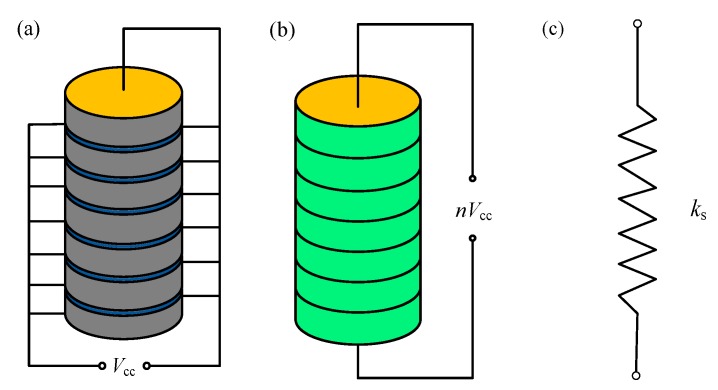
Piezoelectric ceramic structure: (**a**) actual PSA; (**b**) equivalent PSA model; (**c**) equivalent linear spring.

As shown in Figure 9a, the actual PSA is glued by *n* piezoelectric ceramics. There is a glued film and electrode layer between the ceramic. The upper and lower surface of the ceramic is compressed by an electrode. The polarization direction coincides with the axial direction. The material is transversely isotropic. The ceramic only has axial deformation and an electric field. If the effect of the electric field edge and the leakage current is not considered, the one-dimensional constitute equation can be written as:

(3) $$\{\begin{array}{c} \sigma \\ D \end{array}\}=\left[\begin{array}{cc} c^{E} & -e\\ e & \mu^{S} \end{array}\right]\left\{\begin{array}{c} \varepsilon \\ E \end{array}\right\}$$

namely,

(4) $$\sigma =\underset{\mathrm{term}\;1}{\underbrace{c^{E}\varepsilon }}-\underset{\mathrm{term}\;2}{\underbrace{eE}}$$

where σ, ε, and $c^{E}$ are stress, strain, and the elastic coefficients in the axial direction, respectively. *D*, *E*, and *μ*^s^ are the electric displacement, electric field intensity, and dielectric constant, respectively. The second term of Equation (4) is the piezoelectric stress

(5) $$\sigma_{p}=eE$$

where the piezoelectric coefficient $e=E_{p}d$. *d* and *E*_P_ are the piezoelectric constant and Young’s modulus of PSA, respectively.

Each ceramic has the same geometric and physical characteristic and electric load. If the glued film and electrode layer are ignored, the whole piezoelectric stack has a uniform deformation and the displacements assume a linear distribution. If the effect of edge and the discontinuous nature of the electric field are neglected, the electric field is uniform and the potential is a linear distribution. Hence, an equivalent PSA model can be established (see Figure 9b). Based on this model, the electric field intensity can be expressed as $E=V_{\mathrm{cc}}/l_{0}$, where $V_{\mathrm{cc}}$ is the transient voltage applied on PSA and it is a function of voltage amplitude, voltage frequency, and time. *l*_0_ is the thickness of piezoelectric plate layer of the stacked actuator, and the length of the actuator is *nl*_0_, where *n* number of layers of the stacked actuator.

Further, the piezoelectric force can be written as:

(6) $$F_{e}=\sigma_{p}A$$

where *A* is the cross section of the piezoelectric actuator. Substitute Equation (5) into Equation (6), the piezoelectric force is rewritten as:

(7) $$F_{e}=(E_{p}dV_{\mathrm{cc}}/l_{0})A$$

Then, we can regard the model as a liner spring with its stiffness being $k_{s}=E_{p}A/(nl_{0})$ (see Figure 9c). Finally, the piezoelectric force is simplified as:

(8) $$F_{e}=k_{s}ndV_{\mathrm{cc}}=k_{s}qV_{\mathrm{cc}}$$

where q=nd. Here, we can obtain $k_{s}=4.89\times 10^{7}\;N/m$ according to the parameters in the production manual of the piezoelectric stack.

### 3.2. Dynamic Equation during Contact-Impact Phase

**Figure 10:**
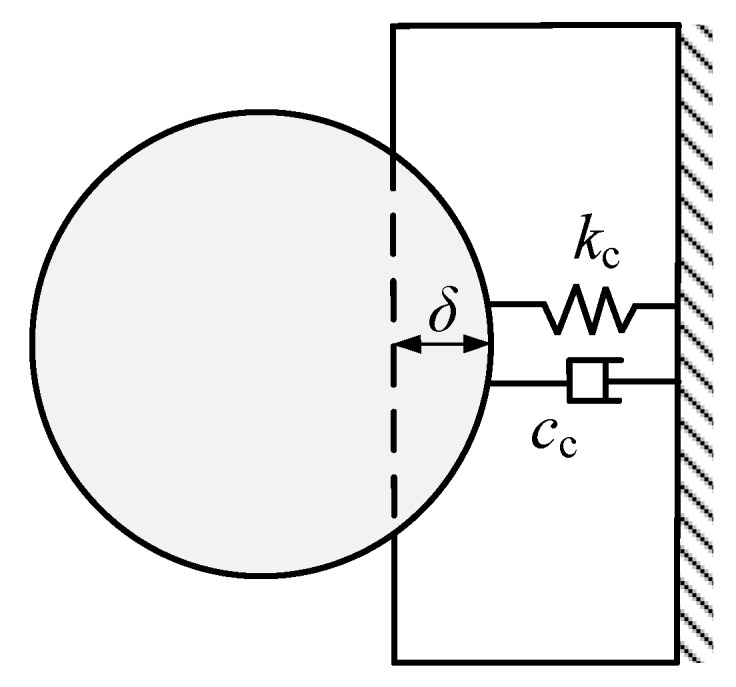
Local viscoelastic contact model between PSA and the object.

For determining the contact force between the PSA and the object, a parallel linear spring-damper element [15] (see Figure 10) is adopted as follows:

(9) $$F_{c}=c_{c}\dot{\delta }+k_{c}\delta$$

where $\delta =X_{1}-X_{2}$ is the indentation between two impact bodies. *X*_2_ is the displacement of the object during the contact-impact phase. $\dot{\delta }$ represents the rate of indentation. $k_{c}$ is a contact stiffness coefficient indicating the ratio of contact force to the indentation. $c_{c}$ is a contact damping coefficient indicating the energy loss due to impact.

For PSA, its dynamic equation with contact constraint (i.e., during contact impact phase) is:

(10) $$m{\ddot{X}}_{1}=-c_{s}{\dot{X}}_{1}-k_{s}X_{1}+F_{e}-F_{c}$$

For the object, its dynamic equation is:

(11) $$M{\ddot{X}}_{2}=F_{c}$$

where *M* is the mass of the object.

By combining Equations (10) and (11), then substituting Equation (9) into them, we get the dynamic equation of the whole driving system during the contact-impact phase before the separation of the object and PSA (i.e., $X_{1}-X_{2}\geq 0$).

(12) $$\frac{dY(t)}{dt}=AY(t)+B(t)$$

where

(13) $$Y(t)={\left[X_{1}{\dot{X}}_{1}X_{2}{\dot{X}}_{2}\right]}^{T}$$

and

(14) $$A=\left[\begin{array}{cccc} 0 & 1 & 0 & 0\\ -\frac{k_{s}+k_{c}}{m} & -\frac{c_{s}+c_{c}}{m} & \frac{k_{c}}{m} & \frac{c_{c}}{m}\\ 0 & 0 & 0 & 1\\ \frac{k_{c}}{M} & \frac{c_{c}}{M} & -\frac{k_{c}}{M} & -\frac{c_{c}}{M} \end{array}\right]$$

(15) $$B(t)={\left[\begin{array}{cccc} 0 & \frac{F_{e}}{m} & 0 & 0 \end{array}\right]}^{T}$$

Once $X_{1}-X_{2}<0$, it means the object separates from PSA. Hence, the contact-impact phase terminates, and it comes into the post-impact phase. Here, the displacement of the object on the end of the contact-impact phase is written as $X_{2}^{0}$, and the initial driving velocity of the object at the beginning of the post-impact phase is defined as ${\dot{X}}_{2}^{0}$.

### 3.3. Dynamic Equation during Post-Impact Phase

The object does not contact the PSA, yet after the contact-impact phase, it undergoes a single pendulum motion with an initial velocity ${\dot{X}}_{2}^{0}$.

The horizontal displacement of the object $X_{2}$ during the post-impact phase can be calculated with the theorem of dynamics. Here, the initial displacement of the object $X_{2}^{0}$ during the contact-impact can be neglected because it is extremely small compared with the large displacement during the post-impact phase. It has little effect for calculating *L*_r_, the maximum of $X_{2}$ during the post-impact phase.

The dynamic equation and the corresponding initial conditions during the post-impact phase are:

(16) $$\left\{ \begin{array}{l} \frac{d^{2}\theta }{dt^{2}}+\frac{g}{l}\sin\theta =0\\ {\theta |}_{t=t_{0}}=0\\ {\dot{\theta }|}_{t=t_{0}}={\dot{X}}_{2}^{0}/l \end{array}\right.$$

where, θ is the swing angle of the object (i.e., the angle between the string and the vertical direction). According to the equation, when $\dot{\theta }\;=\;0$, the maximum of θ written as $\theta_{m}$ can be obtained. Based on the geometry, the following equations are utilized to calculate *L*_r_.

(17) $$\left\{ \begin{array}{l} \cos\theta_{m}=(l-h)/l\\ L_{r}=l\sin\theta_{m} \end{array}\right.$$

where *l* is the length of the string an *h* is the lifted height of the object.

### 3.4. Numerical Solving Strategy

The parameters including *k*_c_, *c*_s_, and *c*_c_ are identified by comparing the experimental data with the solution of the differential Equation (12). In order to solve Equation (12), the fourth-order Runge–Kutta numerical integration method is implemented.

**Figure 11:**
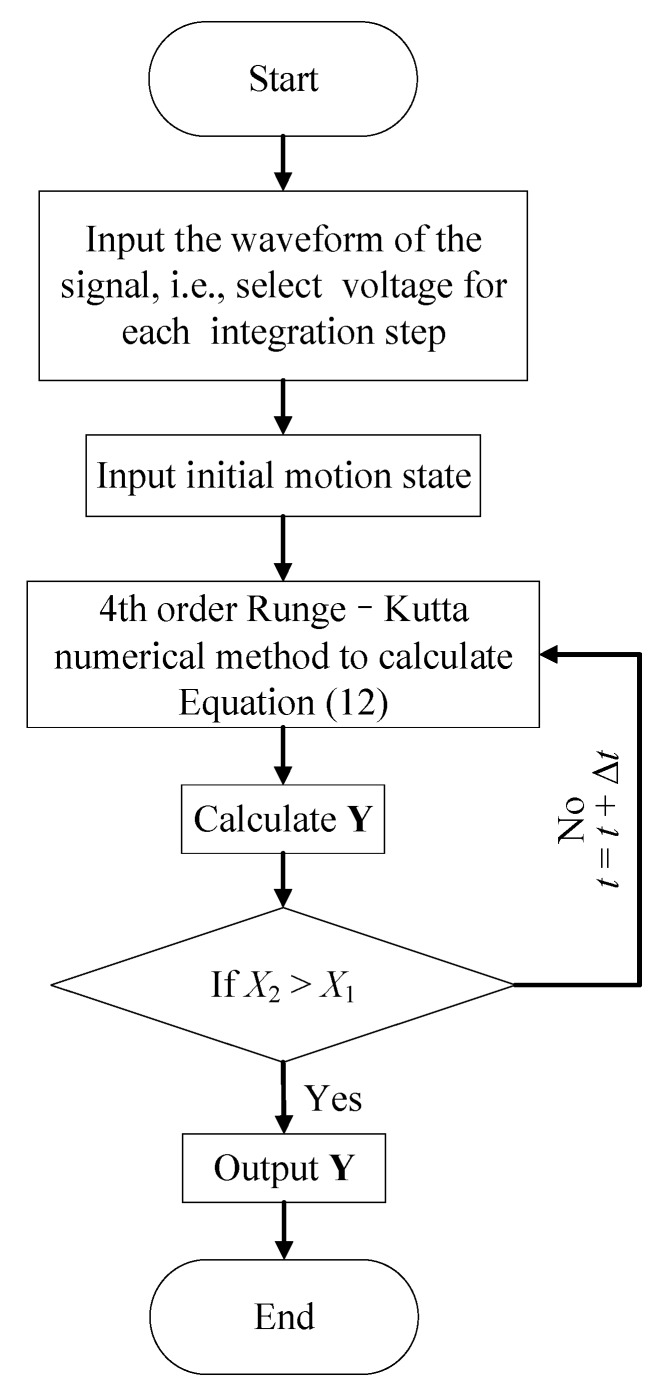
Computational flow.

Figure 11 is the computational flow solving the process. The algorithm can be divided into four parts:

(1) Assign the initial conditions, the terminate conditions, and the total iteration time of the contact-impact phase.
(2) Use the Runge–Kutta numerical integration method to compute the Y(t) in every step.
(3) Determine whether the steel object contacts the PSA or not.
(4) Obtain the initial displacement and the velocity of the object during the post-impact phase.

After the numerical computation during the contact-impact phase, $X_{2}^{0}$ and ${\dot{X}}_{2}^{0}$ when the object separates from PSA is obtained. Then, substituting $X_{2}^{0}$ and ${\dot{X}}_{2}^{0}$ into Equations (16) and (17), we get the launch range *L*_r_.

Through repeating the above strategy, a series of computational solutions are obtained under different amplitudes and frequencies of voltages, which are used to compare the experimental data. During the identification process, the frequency is not set to be a constant. Actually, to finish one identification process, the frequencies we need to use are a series of values (e.g., for the case of *V*_a_ = 20 V, the frequencies change from 0.1 kHz to 25 kHz).

## 4. Numerical Results and Contact Characteristics

In the computation, the waveform is set to be the same as that used in the experiment. The amplitude of the driving voltage is 20 V, and the frequency varies from 1 Hz to 25 kHz. The length of time step Δ*t* is 1 μs with the total time being 80 m. The other system parameters are listed in Table 3. The mass of object *M*, the mass of PSA *m*, *n* times of piezoelectric constant *q*, the length of string *l*, and the stiffness of PSA *k*_s_ are the material or geometry parameters. The values of contact stiffness *k*_c_, structural damping *c*_s_, and contact damping *c*_c_ are achieved by fitting the experimental data under sine wave voltage with *V*_a_ = 20 V (i.e., group 1). The initial conditions of the differential Equation (12) are set to be zeros, namely, $Y(0)={\left[X_{1}{\dot{X}}_{1}X_{2}{\dot{X}}_{2}\right]}^{T}=0$.

### 4.1. Prediction of Launch Range

**Figure 12:**
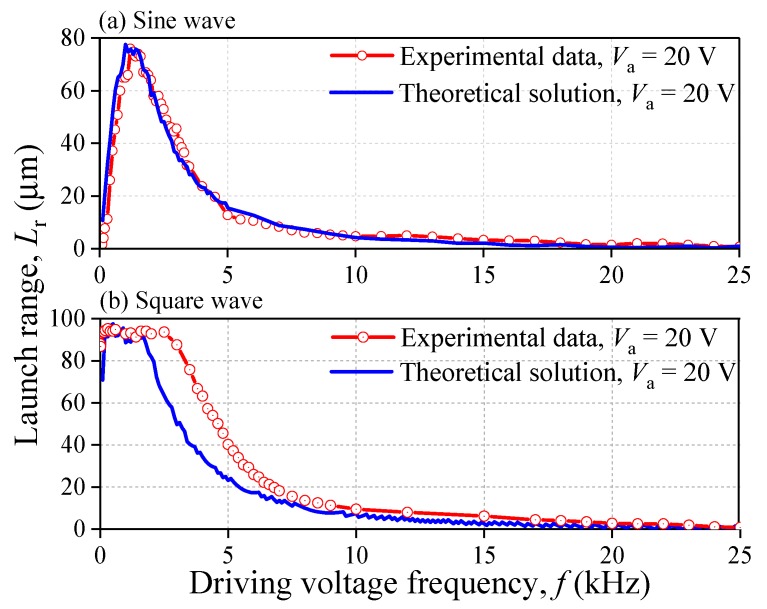
Influence of driving voltage frequency *f* on launch range *L*_r_.

Figure 12 gives the theoretical solution of launch range *L*_r_ under different driving voltages. It shows that the curves of computational solution have a good agreement with that of experimental data. The maximum of *L*_r_ of the experimental data and the theoretical solution are almost the same. In addition, both curves have the same tendency with the increase of *f*. For the sine waveform, the largest *L*_r_ of the theoretical solution is 77.5 μm at 1 kHz while the experimental one is 76 μm at 1.2 kHz. For the square waveform, the largest *L*_r_ of the theoretical solution is 95.5 μm while the experimental one is 95.2 μm. The potential waveform distortion may cause the horizontal segment of the experimental curve to be longer than that of theoretical curve. Table 4 lists some data of *L*_r_ calculated by proposed theoretical method for different amplitude of voltage *V*_a_, sine wave. It shows that the theoretical solutions are also good enough for the different *V*_a_.

### 4.2. Contact Mechanics Behavior and Coefficient of Restitution of Impact

**Figure 13:**
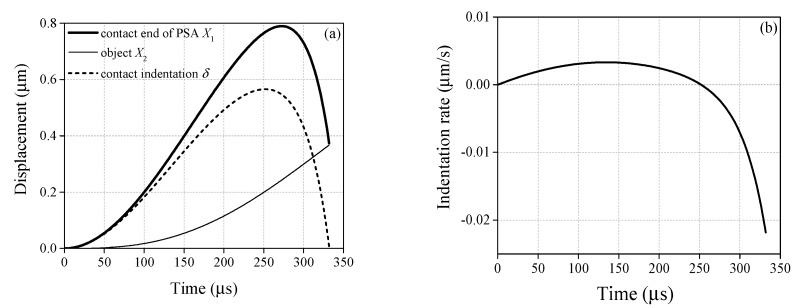
Contact mechanics behavior during the driving process (sine driving voltage, *f* = 1.2 kHz, *V*_a_ = 20 V): (**a**) displacement of the object and the contact end of PSA; (**b**) contact indentation rate.

It is well known that the stack will generate a deformation quickly once the driving voltage is applied. However, the object remains in its former state because of the inertia effect. There exists a contact force (i.e., indentation δ) between the object and PSA. To demonstrate the contact details clearly, the contact indentation and its rate in Equation (9) are calculated. The displacements of the object *X*_2_ and the contact end of PSA *e*_1_ during the contact period are also displayed in Figure 13a. Figure 13b is the indentation rate $\dot{\delta }$. It can be found that *X*_1_ does not increase until roughly 275 μs and then decreases, which is similar to the change of sine driving voltage. On the other hand, *X*_2_ is less than *X*_1_, but it grows up gradually until the object catches up with the contact end of PSA. This phenomenon leads to the displacement difference *X*_1_ – *X*_2_ (i.e., indentation δ, see the short dash line shown in Figure 13a), which can be used to calculate the contact force.

**Figure 14:**
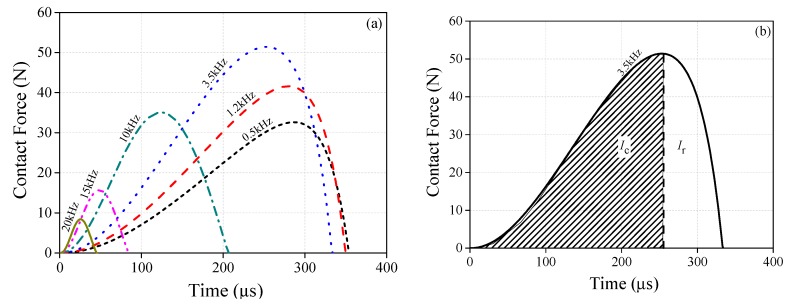
Contact forces during contact-impact: (**a**) time history of contact force; (**b**) schematic of compression and restitution period.

Since *L*_r_ is a frequency-dependent variation, the contact forces should take on a frequency interdependency, which is illustrated in Figure 14. The variation of the contact forces at different frequencies are plotted in Figure 14a. In each curve, the contact force always increases firstly and then decreases as the time goes on. The indentation δ grows at first and then decreases rapidly. Moreover, two variations are defined here in order to specify the change law of the contact forces, namely, $F_{m}$ for the peak value of contact force and $t_{d}$ for the duration of the contact. With the increasing frequency, $t_{d}$ decreases. $F_{m}$ can increase from 0.5 kHz to 3.5 kHz, but it decreases when *f* is higher than 3.5 kHz. Furthermore, the contact impulse imposed on the object is obtained by calculating the area between the curve and the time axis. The change rule of the contact impulse is a coincidence with that of the blue curve plotted in Figure 12a.

**Figure 15:**
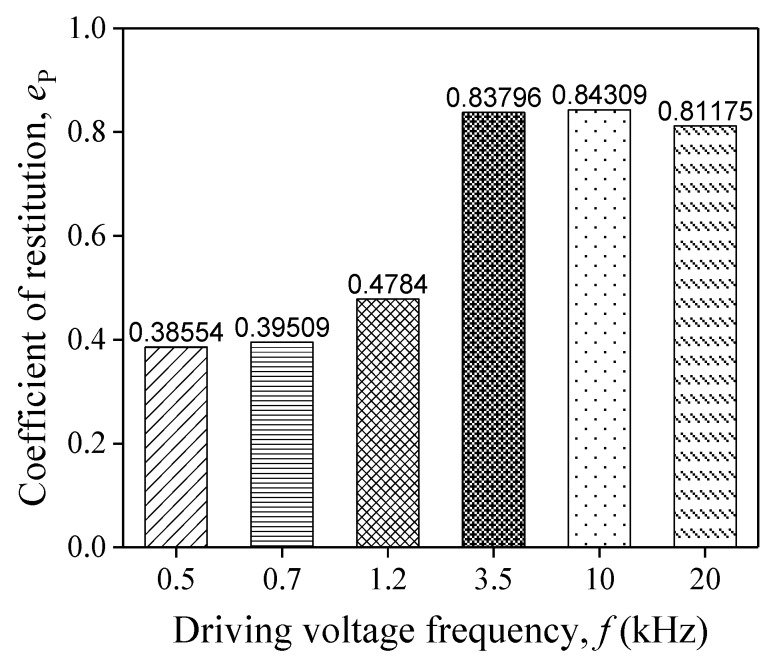
Coefficient of restitution for different *f*.

Figure 14a also shows that there is a single compression–restitution transition during contact. The Poisson’s coefficient of restitution (COR) is defined as

(18) $$e_{P}=\frac{I_{r}}{I_{c}}$$

where $I_{r}$ and $I_{c}$ are the contact impulse during the restitution and compression process. It is easy to obtain these two impulses by integrating the curves as Figure 14b shows. Figure 15 is the histogram of COR. CORs are 0.386, 0.395, and 0.478 when *f* is 0.5 kHz, 0.7 kHz, and 1.2 kHz, respectively. For the case of *f* ≥ 3.5 kHz, their CORs are 0.838, 0.843, and 0.812, which are almost twice as large than at relatively lower frequencies (i.e., f≤1.2 kHz).

In the engineering application of impact dynamics, engineers usually use COR combined with generalized impulse–momentum balance (or other practical engineering methods) to calculate the motion state, including displacement and velocity, after contact-impact. Hence, an accurate value of COR plays a key role in analyzing impact responses of practical piezoelectric driving devices quickly by simple engineering methods. This paper provides the CORs under different driving voltages. These results are convenient for engineers to design piezoelectric devices.

## 5. Conclusions

During the experiment analysis and theoretical computation, some important physical quantities under different diving conditions are obtained including the launch ranges *L*_r_ of the object, the contact forces, and the coefficients of restitution. The results show that the computational solution has a good agreement with the experimental data. Driven by a 20 V sine wave, the error of the largest *L*_r_ between the experimental data and the computational solution is only 1.97% as the frequency *f* of the driving voltage varies. The curve illustrating the relationship between *f* of the driving voltage and *L*_r_ reaches a peak value at around 1.2 kHz. In addition, by using the proposed theoretical method, the contact force solutions show that as *f* increases, the peak value of the contact force increases firstly and decreases subsequently, with a maximum that appears at 3.5 kHz. The CORs are roughly 0.5 when *f <* 3.5 kHz as well as roughly 0.9 when *f* ≥ 3.5 kHz. 

## Figures and Tables

**Table 1:** The system parameters of experimental setup.

PSA		LDV		High-Speed Camera	
M	NEC\TOKIN AE0505D16DF	M	OFV-5000	M	MEMRECAM GX-3
SC	1.5 μF	WD	0.5~100 m	ST	CCD
RF	69 kHz	*v*_max_	10 (m/s)	R	45
Stiffness	48.9 (N/μm)	FR	DC~24 MHz		
		VR	Over 0.01 (μm/s)		
		DR	Over 0.05 pm		

Notes: LDV: laser doppler vibrometer; M: product model; SC: static capacity; RF: resonance frequency; WD: working distance; *v*_max_: maximum velocity; FR: frequency range; VR: velocity resolution; DR: displacement resolution; ST: sensor type; R: resolution; CCD: charge-coupled device.

**Table 2:** Experimental scheme.

Group	Waveform	Amplitude (V)	Frequency (kHz)	Launch Range (μm)
1	Sine wave	20	0.1–25	0.67–76.00
2	Sine wave	15	0.1–18	0.90–34.2
3	Sine wave	1–20	1.2	0.37–76.00
4	Sine wave	3–20	3.0	0.4–34.4
5	Square wave	1–20	1.2	0.09–93.2
6	Square wave	20	0.001–50	0–94.8

**Table 3:** System parameters using in computation (unit: SI).

*M*	*m*	*q*	*l*	*k*_s_	*k*_c_	*c*_s_	*c*_c_
0.027	0.005	1.2 × 10^−7^	0.09	4.89 × 10^7^	1.425 × 10^5^	13,991	68

Notes: SI refers to International System (used to describe units of measurement; from French Système International).

**Table 4:** Data of experimental and the theoretical solution of *L*_r_ (sine wave).

*V*_a_ (V)	*f* = 1.2 kHz		*f* = 3.0 kHz	
	Theoretical *L*_r_ (μm)	Experimental *L*_r_ (μm)	Theoretical *L*_r_ (μm)	Experimental *L*_r_ (μm)
16	60.60	46.40	28.97	18.40
17	64.40	52.00	30.70	21.20
18	68.18	59.20	32.60	24.40
19	72.96	68.40	34.41	27.70
20	75.75	76.00	36.22	34.40

## Appendix A

**Table A1:** (**a**) Data in Figure 4. Sine Wave, *f* = 1.2 kHz. (**b**) Data in Figure 4. Sine Wave, *f* = 3 kHz.

(**a**)							
***V*_a_ (V)**	***L*_r_ (μm)**	***V*_a_ (V)**	***L*_r_ (μm)**	***V*_a_ (V)**	***L*_r_ (μm)**	***V*_a_ (V)**	***L*_r_ (μm)**
20.00	76.00	14.50	33.60	9.00	9.00	3.50	0.93
19.50	71.00	14.00	30.00	8.50	7.70	3.00	0.90
19.00	68.40	13.50	28.00	8.00	6.80	2.00	0.37
18.50	66.00	13.00	27.20	7.50	5.30	1.00	0.44
18.00	59.20	12.50	23.20	7.00	4.70		
17.50	54.40	12.00	20.80	6.50	4.36		
17.00	52.00	11.50	18.00	6.00	3.36		
16.50	49.60	11.00	16.40	5.50	2.56		
16.00	46.40	10.50	15.20	5.00	2.08		
15.50	43.20	10.00	14.40	4.50	1.56		
15.00	36.80	9.50	11.60	4.00	1.32		
(**b**)							
***V*_a_ (V)**	***L*_r_ (μm)**	***V*_a_ (V)**	***L*_r_ (μm)**	***V*_a_ (V)**	***L*_r_ (μm)**	***V*_a_ (V)**	***L*_r_ (μm)**
20.00	34.40	15.50	16.80	11.00	6.05	6.50	2.18
19.50	33.20	15.00	15.40	10.50	4.89	6.00	1.76
19.00	27.70	14.50	13.80	10.00	4.48	5.50	1.52
18.50	27.60	14.00	13.60	9.50	4.34	5.00	1.21
18.00	24.40	13.50	12.00	9.00	3.66	4.50	1.03
17.50	22.00	13.00	10.20	8.50	3.06	4.00	0.85
17.00	21.20	12.50	8.50	8.00	3.31	3.50	0.57
16.50	19.40	12.00	7.50	7.50	3.32	3.00	0.40
16.00	18.40	11.50	6.70	7.00	2.62		

**Table A2:** (**a**) Data in Figure 5. Sine Wave, *V*_a_ = 20 V. (**b**) Data in Figure 5. Sine Wave, *V*_a_ = 15 V.

(**a**)									
***f* (Hz)**	***L*_r_ (μm)**	***F* (Hz)**	***L*_r_ (μm)**	***f* (Hz)**	***L*_r_ (μm)**	***f* (Hz)**	***L*_r_ (μm)**	***f* (Hz)**	***L*_r_ (μm)**
100.00	1.64	1500.00	74.00	3000.00	45.60	8500.00	5.86	22,000.00	1.90
150.00	4.00	1600.00	73.00	3100.00	40.50	9000.00	5.32	23,000.00	1.45
200.00	7.70	1700.00	67.00	3200.00	38.50	9500.00	5.00	24,000.00	0.89
300.00	11.30	1800.00	67.00	3300.00	36.50	10,000.00	4.66	25,000.00	0.67
400.00	26.00	1900.00	66.00	3400.00	31.70	11,000.00	4.74		
500.00	37.20	2000.00	64.00	3500.00	31.20	12,000.00	4.96		
600.00	45.20	2100.00	58.00	4000.00	23.70	13,000.00	4.52		
700.00	50.80	2200.00	56.00	4500.00	19.70	14,000.00	3.84		
800.00	60.00	2300.00	58.00	5000.00	12.70	15,000.00	3.26		
900.00	65.00	2400.00	55.10	5500.00	11.05	16,000.00	3.08		
1000.00	65.00	2500.00	53.00	6000.00	10.55	17,000.00	2.98		
1100.00	66.00	2600.00	49.00	6500.00	9.32	18,000.00	2.30		
1200.00	76.00	2700.00	46.50	7000.00	8.32	19,000.00	1.64		
1300.00	74.80	2800.00	46.00	7500.00	6.92	20,000.00	1.44		
1400.00	73.00	2900.00	44.50	8000.00	6.08	21,000.00	2.01		
(**b**)									
***f* (Hz)**	***L*_r_ (μm)**	***f* (Hz)**	***L*_r_ (μm)**	***f* (Hz)**	***L*_r_ (μm)**	***f* (Hz)**	***L*_r_ (μm)**		
100.00	0.90	1500.00	33.20	3000.00	16.80	8500.00	2.68		
150.00	2.04	1600.00	32.20	3100.00	16.40	9000.00	2.28		
200.00	3.84	1700.00	31.40	3200.00	15.60	9500.00	2.12		
300.00	7.84	1800.00	30.20	3300.00	14.40	10,000.00	1.90		
400.00	12.80	1900.00	28.80	3400.00	13.80	11,000.00	1.94		
500.00	16.40	2000.00	26.80	3500.00	14.20	12,000.00	2.08		
600.00	22.00	2100.00	26.80	4000.00	11.20	13,000.00	1.72		
700.00	25.80	2200.00	25.40	4500.00	8.44	14,000.00	1.36		
800.00	28.80	2300.00	25.00	5000.00	5.92	15,000.00	1.12		
900.00	30.40	2400.00	24.20	5500.00	4.76	16,000.00	1.10		
1000.00	32.20	2500.00	24.40	6000.00	3.48	17,000.00	1.12		
1100.00	33.20	2600.00	22.60	6500.00	3.80	18,000.00	0.90		
1200.00	34.20	2700.00	21.40	7000.00	3.04				
1300.00	33.60	2800.00	19.80	7500.00	2.64				
1400.00	33.60	2900.00	18.40	8000.00	2.88				

**Table A3:** Data in Figure 6. Square wave, *f* = 1.2 kHz.

*V*_a_ (V)	*L*_r_ (μm)	*V*_a_ (V)	*L*_r_ (μm)	*V*_a_ (V)	*L*_r_ (μm)	*V*_a_ (V)	*L*_r_ (μm)
20.00	93.20	15.00	48.85	10.00	18.00	5.00	3.36
19.50	89.60	14.50	44.40	9.50	14.60	4.50	2.80
19.00	85.20	14.00	40.40	9.00	13.50	4.00	2.16
18.50	78.40	13.50	37.20	8.50	11.70	3.50	1.60
18.00	74.80	13.00	32.40	8.00	10.60	3.00	1.12
17.50	69.20	12.50	30.10	7.50	9.30	2.50	0.74
17.00	63.60	12.00	26.40	7.00	7.60	2.00	0.40
16.50	60.00	11.50	23.40	6.50	7.30	1.50	0.20
16.00	55.20	11.00	20.60	6.00	5.00	1.00	0.09
15.50	50.40	10.50	19.60	5.50	4.32		

**Table A4:** Data in Figure 7. Square wave, *V*_a_ = 20 V.

*f* (Hz)	*L*_r_ (μm)	*f* (Hz)	*L*_r_ (μm)	*f* (Hz)	*L*_r_ (μm)	*f* (Hz)	*L*_r_ (μm)
1.00	88.00	1800.00	94.00	5800.00	29.40	18,000.00	4.08
10.00	88.00	2000.00	92.80	6000.00	26.00	19,000.00	3.45
20.00	86.80	2500.00	93.60	6200.00	24.80	20,000.00	2.76
50.00	93.00	3000.00	87.60	6400.00	22.20	21,000.00	2.56
100.00	93.60	3500.00	75.80	6600.00	21.20	22,000.00	2.52
150.00	93.60	3800.00	66.80	6800.00	19.80	23,000.00	1.90
200.00	94.40	4000.00	63.20	7000.00	18.20	24,000.00	1.16
300.00	95.20	4200.00	57.20	7500.00	15.60	25,000.00	0.85
400.00	94.00	4400.00	54.00	8000.00	13.60	30,000.00	0.47
500.00	94.00	4600.00	50.20	8500.00	12.50	40,000.00	0.09
600.00	94.80	4800.00	45.60	9000.00	11.30	50,000.00	0.00
1000.00	92.80	5000.00	40.20	10,000.00	9.50		
1200.00	93.20	5200.00	37.20	12,000.00	8.00		
1400.00	91.30	5400.00	34.00	15,000.00	6.24		
1600.00	94.00	5600.00	30.60	17,000.00	4.48		
